# Conventional Treatments plus Acupuncture for Asthma in Adults and Adolescent: A Systematic Review and Meta-Analysis

**DOI:** 10.1155/2019/9580670

**Published:** 2019-01-17

**Authors:** Chunxiang Jiang, Lanlan Jiang, Qingwu Qin

**Affiliations:** ^1^Department of Pulmonary and Critical Care Medicine, The Second Xiangya Hospital, Central South University, Changsha, Hunan 410011, China; ^2^Department of Pulmonary and Critical Care Medicine, Xiangya Hospital, Central South University, Changsha, Hunan 410008, China; ^3^Hunan Diagnosis and Treatment Center of Respiratory Disease, Changsha, Hunan 410011, China; ^4^Research Unit of Respiratory Disease, Central South University, Changsha, Hunan 410011, China

## Abstract

**Objectives:**

The efficacy of conventional treatments plus acupuncture for asthma in adult and adolescent is uncertain. Literature reports are conflicting; therefore, the aim of this study was to determine the efficacy of conventional treatments plus acupuncture versus conventional treatments alone using a meta-analysis of all published randomized clinical trials (RCTs).

**Methods:**

Two reviewers independently performed a comprehensive literature search from multiple electronic sources (1990–2018), including PubMed, EMBASE, the Cochrane Library, Web of Science, China National Knowledge Infrastructure, and WanFang databases. RCTs in which adult and adolescent patients with asthma (age ≥12 years) were divided into conventional treatments plus acupuncture and conventional treatments alone were included.

**Results:**

Nine studies were included. The results showed that conventional treatments plus acupuncture as a complementary therapy could improve the symptom response rate (OR = 7.87, 95% CI = [4.13, 14.99], p < 0.00001) and significantly decrease interleukin-6 (IL-6) levels (MD = -11.42; 95% CI = [-15.28, -7.56], p < 0.00001). However, indices of pulmonary function, including the forced expiratory volume in one second (FEV_1_) (MD = 0.22, 95% CI = [-0.11, 0.56], p = 0.19) and FEV_1_/forced vital capacity (FVC) (MD = 8.62, 95% CI = [-0.35, 17.59], p = 0.06), failed to be improved with conventional treatments plus acupuncture.

**Conclusion:**

Conventional treatments plus acupuncture are associated with significant benefits for adult and adolescent patients with asthma. Therefore, we suggest the use of conventional treatments plus acupuncture for asthma patients.

## 1. Introduction

Asthma is one of the most common chronic diseases with a multifactorial etiology that is characterized by chronic airway inflammation, hyperresponsiveness, and reversible airway obstruction, affecting approximately 358 million people worldwide [1–3]. Over the past few decades, the global prevalence, mortality, morbidity, and economic burden of asthma have increased [3–10]. The treatment response for a particular patient may be difficult to predict due to the heterogeneous nature of asthma [11, 12].

Despite the availability of clinical practice guidelines and standard-of-care therapy, a large proportion of asthma patients have a poor quality-of-life [13, 14]. Meanwhile, the majority of the currently available therapeutic approaches, such as muscle relaxants and anti-inflammatory drugs, cannot change the natural course of asthma [15].

Therefore, a high unmet requirement for novel asthma therapeutics is necessary, especially for those with severe disease. Many asthma patients turn to complementary or alternative therapies to improve their conditions. In China, acupuncture has been used to manage asthma for thousands of years. Asthma was listed as an indication for acupuncture in 1979 by the World Health Organization (WHO) [16] and has been recommended as an adjunctive treatment in comprehensive management programs for asthma by the US National Institutes of Health.

Several clinical trials have reported both beneficial and detrimental effects from acupuncture for the treatment of asthma [17–19]. This may be caused by differences in trial design, treatment mode, or the small size of the trials. Previous systematic reviews [20] and meta-analyses have reported the effect of acupuncture. However, most of the studies included randomized clinical trials (RCTs) comparing real and sham acupuncture in asthma patients, which has little instructive significance for clinical treatment as acupuncture is often used as a complement to conventional therapy.

Therefore, in this meta-analysis, RCTs comparing conventional treatments plus acupuncture with conventional treatments alone in asthma patients were included to determine the effect of acupuncture as a supportive therapy.

## 2. Methods and Materials

### 2.1. Search Strategy

Two reviewers independently performed a comprehensive literature search from multiple electronic sources, including PubMed, EMBASE, the Cochrane Library, Web of Science, China National Knowledge Infrastructure, and the WanFang databases between Jan. 1, 1990, and June 1, 2018. The following search terms were used: (acupuncture therapy, acupuncture, needling therapy, needling) and (bronchial asthma, asthma, asthmatic) to identify relevant papers. The terms were combined appropriately with medical subheading terms and appropriately adjusted for different databases. For example, (((((((acupunture therapy) OR acupuncture) OR needling therapy) OR needling) OR “Acupunture Therapy” [MeSH]))) AND ((((bronchial asthma) OR asthma) OR asthmatic)) AND (((“Randomized Controlled Trial” [Publication Type] OR “Randomized Controlled Trials as Topic” [MeSH])) OR random*∗*))) Filters: Publication date from 1990/01/01 to 2018/06/01. The detailed search strategy is provided in supplementary materials (available here).

### 2.2. Study Selection

#### 2.2.1. Inclusion Criteria for Studies

(1) RCTs were published in English or Chinese, with the full text available.

(2) Included patients met the diagnostic criteria for bronchial asthma.

(3) RCTs compared conventional treatments plus acupuncture with conventional treatments alone.

(4) Studies measured at least one of the following end points: symptom response rate, forced expiratory volume in one second (FEV1), FEV_1_/forced vital capacity (FVC), and interleukin-6 (IL-6) levels.

#### 2.2.2. Exclusion Criteria for Studies

(1) Studies only involved cells or animals or were case reports, letters, reviews, observational studies, or nonrandomized clinical trials.

(2) Included patients were children younger than 12 years of age.

(3) Trials in which acupuncture therapies were used with other therapies.

#### 2.2.3. Data Extraction and Management

The extraction table was designed according to the Cochrane manual. Identified publications from databases were independently screened by two authors to exclude the irrelevant studies. Controversial opinions were resolved by discussion.

### 2.3. Bias Assessment of the Included Studies

Risk of bias was assessed using the Cochrane classification for the following seven criteria: random sequence generation, allocation concealment, blinding of participants and personnel, blinding of outcome assessment, incomplete outcome data, selective reporting, and other bias.

### 2.4. Statistical Analysis

The data were analyzed using Revman 5.3 and Stata 14.0 software. The results were presented as the mean difference (MD) or odds ratio (OR) and 95% confidence intervals (95% CI). To analyze data without obvious heterogeneity (p > 0.1 and* I*^2^ < 50%) the fixed-effects model was used; otherwise, the random-effects model was used. If possible, heterogeneity was investigated and subgroup analysis was conducted. Sensitivity analysis was also performed to evaluate the influence of an individual study on the final effect. To evaluate publication bias, Egger's test was used. A p value < 0.05 was considered statistically significant.

## 3. Results

### 3.1. Literature Selection

**Figure 1:**
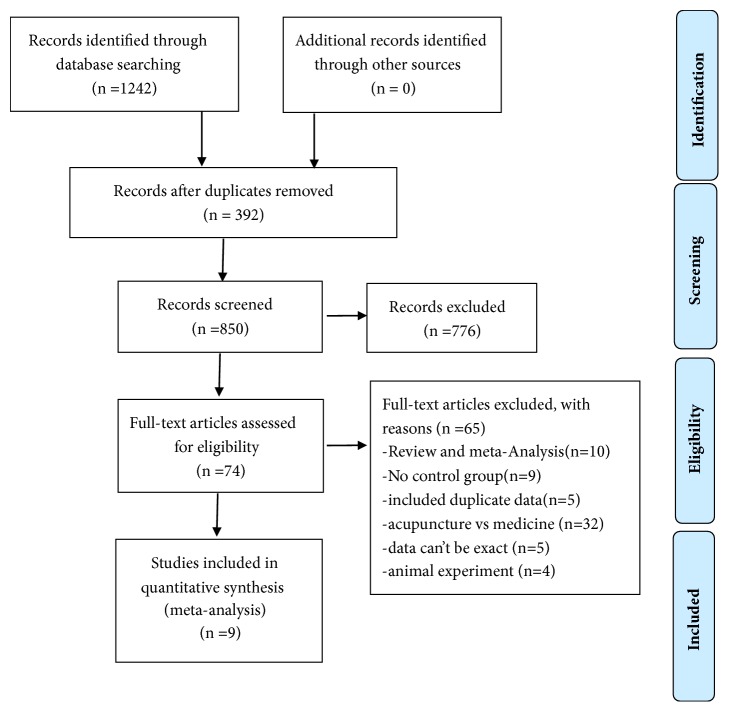
Flow chart for study selection.

The flow diagram of the literature identification and selection process is shown in Figure 1. A total of 1242 studies were identified, and 392 studies were excluded owing to duplication. After reading the titles and abstracts, 776 studies were excluded. The full-length texts of 74 candidate studies were carefully reviewed, of which 65 studies were excluded, including 10 reviews/meta-analyses, 9 studies without a control group, 5 studies with duplicated data, 32 studies involving acupuncture versus medicine, 5 studies with data that could not be extracted, and 4 studies only including animal experiments. Finally, nine trials were included in the quantitative analysis. The characteristics of the nine included trials are shown in Table 1.

### 3.2. Qualitative Assessment of the Included Studies

**Figure 2:**
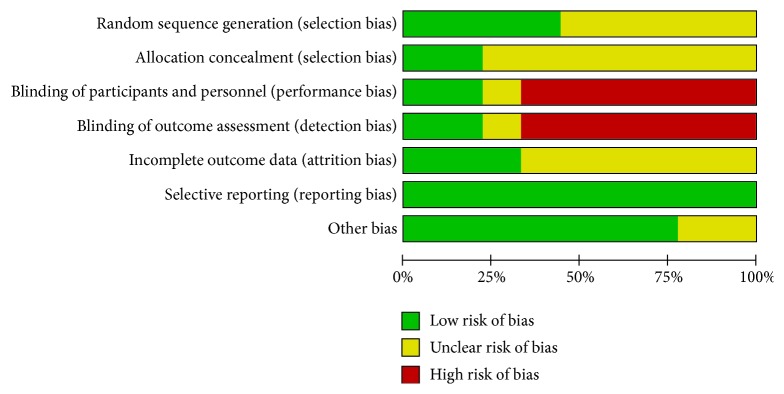
Risk of bias.

**Figure 3:**
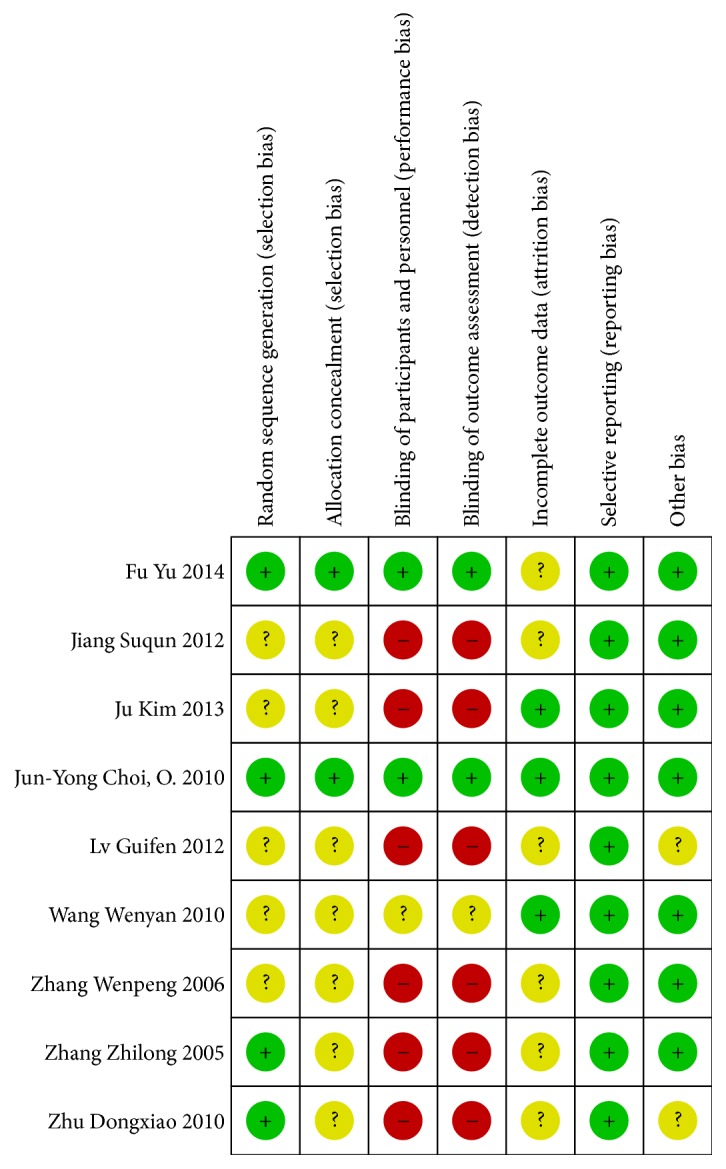
Summary of risk bias.

Among the 9 studies, three studies [21–23] used random number table and one study [24] took computer program to generate random sequence. The remaining 5 studies only mentioned “random” or “randomization”. As it is very difficult to blind therapists to the use of acupuncture therapy, only patients and assessors were blinded. Only two studies [21, 24] reported the allocation concealment and blinding details. In addition, all the included studies had low risk of reporting bias. The results of bias are shown in Figures 2 and 3.

### 3.3. Response Rate

**Figure 4:**
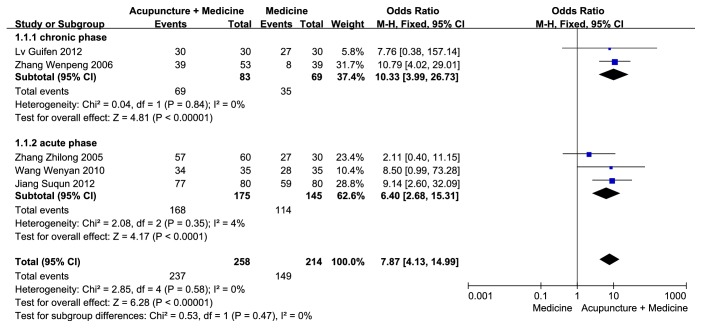
Efficacy of conventional treatments plus acupuncture on the response rate.

Among all nine included studies, five RCTs [22, 25–28] (including 472 patients) reported the response rates of the patients in the two groups. There was no significant heterogeneity (p = 0.58,* I*^2^ = 0%) among these RCTs, and our pooled results showed statistically significant effects of conventional treatments plus acupuncture compared with conventional treatments alone (OR = 7.87, 95% CI = [4.13, 14.99], p < 0.00001) (Figure 4). Subgroup analyses also showed beneficial effects of conventional treatments plus acupuncture both in the chronic phase (OR = 10.33, 95% CI = [3.99, 26.73], p < 0.00001, heterogeneity: p = 0.84,* I*^2^ = 0%) and in the acute phase (OR = 6.40, 95% CI = [2.68, 15.31], p < 0.0001, heterogeneity: p = 0.35,* I*^2^ = 4%).

### 3.4. Parameters of Pulmonary Ventilation

#### 3.4.1. FEV_1_

**Figure 5:**
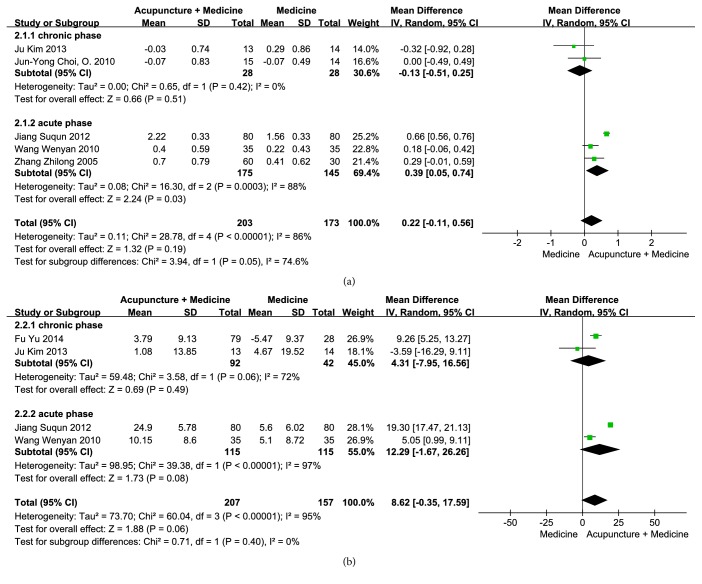
Efficacy of conventional treatments plus acupuncture on (a) FEV_1_ and (b) FEV_1_/FVC.

Five RCTs [22, 24, 27–29] (including 376 patients) compared the effects of conventional treatments plus acupuncture and the conventional treatments alone on FEV_1_. There was significant heterogeneity among these RCTs (p < 0.00001, I^2^ = 86%), a random-effects model was used, and the pooled data showed no difference in therapeutic effects on FEV_1_ between the two groups (MD = 0.22, 95% CI = [-0.11, 0.56], p = 0.19) (Figure 5(a)). Subgroup analyses also showed no improvements in FEV_1_ with acupuncture plus conventional therapy in the chronic phase (MD = -0.13, 95% CI = [-0.51, 0.25], p = 0.51, heterogeneity: p = 0.42,* I*^2^ = 0%). However, the FEV_1_ was improved with acupuncture plus conventional therapy in the acute phase (MD = 0.39, 95% CI = [0.05, 0.74], p = 0.03, heterogeneity: p = 0.0003,* I*^2^ = 88%).

#### 3.4.2. FEV_1_/FVC

The effects between conventional treatments plus acupuncture and conventional treatments alone were investigated in four studies [21, 27–29], including 364 patients. The meta-analysis of these four RCTs failed to show favorable effects of conventional treatments plus acupuncture on FEV_1_/FVC compared with the conventional treatments alone (MD = 8.62, 95% CI = [-0.35, 17.59], p = 0.06) (Figure 5(b)). Marked heterogeneity was observed in this mode (p < 0.00001,* I*^2^ = 95%); therefore, the subgroups were further analyzed. The results showed no improvements in FEV_1_/FVC with acupuncture plus conventional therapy in either the chronic phase (MD = 4.31; 95% CI = [-7.95, 16.56], p = 0.49, heterogeneity: p = 0.06,* I*^2^ = 72%) or in the acute phase (MD = 12.29, 95% CI = [-1.67, 26.26], p = 0.08, heterogeneity: p < 0.00001,* I*^2^ = 97%).

### 3.5. Cytokine Expression

#### 3.5.1. IL-6

**Figure 6:**
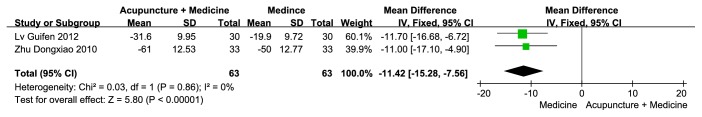
Efficacy of conventional treatments plus acupuncture on IL-6 levels.

Two RCTs [23, 26] compared the effects of conventional treatments plus acupuncture and conventional treatments alone on IL-6 levels. No heterogeneity was found (p = 0.86,* I*^2^ = 0%), and the fixed-effects model showed that the IL-6 level was significantly reduced with conventional treatments plus acupuncture (MD = -11.42, 95% CI = [-15.28, -7.56], p < 0.00001) (Figure 6).

### 3.6. Sensitivity Analysis and Publication Bias

Sensitivity analysis was also performed to evaluate the stability of the results. As fewer than three data sets were identified for IL-6 analysis, a different model was used for its sensitivity analysis, and the results were found to be stable. To test the sensitivity of parameters with more than three data sets, we excluded the included studies one by one, and the results were found to be stable. In addition, Egger's test was used for publication bias analysis, which showed no publication bias (Tables 3 and 4).

## 4. Discussion

Acupuncture has been applied to treat asthma for thousands of years in China. Asthma was listed as an indication for acupuncture in 1979 by the WHO [16]; however, despite its use for millennia, no strong evidence has demonstrated the beneficial efficiency of conventional treatments plus acupuncture compared with conventional treatments alone. Previous research has indicated that acupuncture downregulates the expression of proinflammatory proteins and upregulates the expression of anti-inflammatory proteins [30]. In our current meta-analysis, we pooled the data from nine studies together and demonstrated that conventional treatments plus acupuncture significantly improved the symptom response rate (OR = 7.87, 95% CI = [4.13, 14.99], p < 0.00001) and decreased IL-6 levels (MD = -11.42; 95% CI = [-15.28, -7.56], p < 0.00001), compared with conventional treatments alone. However, ventilation parameters including FEV_1_ (MD = 0.22, 95% CI = [-0.11, 0.56], p = 0.19) and FEV_1_/FVC (MD = 8.62, 95% CI = [-0.35, 17.59], p = 0.06) showed no improvements with conventional treatments plus acupuncture.

We have made efforts to perform a comprehensive analysis; however, some limitations must be recognized. First, significant heterogeneity was found for FEV_1_ and FEV_1_/FVC. Therefore, after checking the included papers and extracted data, subgroup analysis by asthma phase was used to explore the source of heterogeneity. However, subgroup analysis found that combinations of different phases of asthma had no influence on the heterogeneity. Thus, a random-effects model was used for FEV_1_ and FEV_1_/FVC. Furthermore, sensitivity analysis was performed by excluding the included studies one by one, which showed that all parameters were stable. Second, only two studies including 126 patients compared the effects of IL-6 between the two groups; the sample size was too small. Fortunately, we found stable results, assuming a different model to test the sensitivity.

To the best of our knowledge, this is the first meta-analysis that has systematically estimated the effectiveness of conventional treatments plus acupuncture compared with conventional treatments alone. Conventional treatments plus acupuncture did not show any beneficial effects on pulmonary function in asthmatic patients over conventional treatments alone. However, conventional treatments plus acupuncture did show a clinically and statistically significant improvement in the symptom response rate and the IL-6 level when compared with conventional treatments alone. Thus, acupuncture has some effects on asthma, especially as an adjunctive therapy.

Further RCTs are required to confirm that acupuncture treatment has beneficial effects on alleviating symptoms and immunomodulatory activity (via inflammatory cells and cytokines) in asthma patients as a supportive therapy combined with conventional treatments. A standard method of conventional treatments plus acupuncture may be implemented in the near future.

## Figures and Tables

**Table 1:** Characteristics of the included studies.

First author (year)	Sample size	Stage, levels of severity	Sex (F:M)	Study location	Patient age/ Duration of disease	Intervention	Control	Study design
Fu Yu (2014)	107	Chronic persistent, middle	A: 54:25 B: 18:10	Beijing, China	A: 51.82 ± 9.90 y B: 52.40 ± 9.18 y	Ventolin inhalant + Acupuncture (12 w)	Ventolin inhalant (12 w)	A1 vs. A2 vs. A3 vs. B

Jiang Suqun (2012)	160	Acute	A: 37:43 B: 35:45	Hunan, China	A: 32.31 ± 9.32 y B: 34.21 ± 8.33 y	Bronchodilator + ICS + Antibiotics + Acupuncture (14 d) *∗*	Bronchodilator + ICS + Antibiotics (14 d) *∗*	A vs. B

Ju Kim (2013)	27	Chronic persistent	A: 8:5 B: 3:11	Jiangsu, China	A: 52.62 ± 14.16 y B: 57.14 ± 12.24 y	Ventolin inhalant + Acupuncture (12 w)	Ventolin inhalant (12 w)	A vs. B

Jun-Yong Choi (2010)	30	Chronic persistent	A: 7:8 B: 7:8	Korea	A: 48.27 ± 7.99 y B: 50.43 ± 12.64 y	Western medicine + Acupuncture (4 w)*∗*	Western medicine (4 w)*∗*	A vs. B vs. C

Lv Guifen (2012)	90	Chronic persistent	A: 17:13 B: 17:13	Shandong, China	A: 15–68 y B: 16–17 y	Salmeterol Xinafoate and Fluticasone Propionate Powder for Inhalation (50/250 *µ*g, Bid) + Acupuncture (4 w)	Salmeterol Xinafoate and Fluticasone Propionate Powder for Inhalation (50/250 *µ*g, Bid, 4 w)	A vs. B vs. C

Wang Wenyan (2010)	70	Acute	A: 17:18 B: 18:17	Jilin, China	A: 15–65 y/2–15 y B: 14–69 y/2–13 y	Bronchodilator + ICS + Antibiotics + Acupuncture (10 d)*∗*	Bronchodilator + ICS + Antibiotics (10 d)*∗*	A vs. B

Zhang Wenpeng (2006)	104	Chronic persistent	A: 44:15 B: 33:12	Russia	A: 44.5 ± 13.9 y B: 44.2 ± 14.6 y	Western medicine + Acupuncture (12 d)*∗∗*	Western medicine (12 d)*∗∗*	A vs. B

Zhu Dongxiao (2010)	99	Chronic persistent	A: 18:15 B: 20:13	Henan, China	A: 12–85 y B: 12–86 y	Beclomethasone Dipropionate Inhaler + Theophylline Sustained Release Tablets + Acupuncture *∗∗∗*	Beclomethasone Dipropionate Inhaler + Theophylline Sustained Release Tablets *∗∗∗*	A vs. B vs. C

Zhang Zhilong (2005)	90	Acute	A: 32:28 B: 16:14	Tianjin, China	A: 20–65 y/6–32 y B: 19–65 y/5–33 y	Terbutaline sulphate aerosol + Acupuncture (10 d)	Terbutaline sulphate aerosol (10 d)	A vs. B

A: conventional treatments plus acupuncture; B: conventional treatments alone; Western medicine: any drug that is recommended by the Global Initiative For Asthma (GINA), including short-acting beta2-agonist (SABA), theophylline, leukotriene receptor antagonist (LTRA), long-acting beta2-agonist (LABA), inhaled corticosteroids (ICS), and ICS/LABA.

*∗*Specific drugs were not specified.

*∗∗*The details are listed in Table 2.

**Table 2:** Comparison of drugs used between the two groups.

Group	No drug	Cromolyn Sodium	Ventolin	Cromolyn Sodium + Ventolin	Aminophylline + Ventolin	Atrovent	Fenoterol	Beclomethasone Dipropionate	Beclomethasone Dipropionate + Ventolin	Beclomethasone Dipropionate + Fenoterol	Beclomethasone Dipropionate + Aminophylline
Intervention group	3	5	6	3	2	1	9	6	12	9	3

Control group	3	4	6	3	2	1	6	4	8	6	2

Note: the doses of the drugs shown in the table are as follows: Cromolyn Sodium, 10 mg, four times per day (Qid); Ventolin, 0.1 mg, Qid; Fenoterol, 0.2 mg, three times per day (Tid); Beclomethasone Dipropionate, 250 mg, Tid; Aminophylline, 0.1 g, Tid; Atrovent, 40 *µ*g, Tid.

*∗∗∗*The course of treatment was not specified.

**Table 3:** Summary of sensitivity analysis for the response rate.

	OR Fluctuation	95% CI Fluctuation	Publication bias (P value)
Response rate	3.93–7.73	1.90–14.85	0.806

Note: p < 0.05 indicates that a publication bias exists.

**Table 4:** Summary of sensitivity analysis of parameters for FEV_1_ and FEV_1_/FVC.

	MD Fluctuation	95% CI Fluctuation	Publication bias (P value)
FEV_1_	0.14–0.32	-0.23–0.65	0.144
FEV_1_/FVC	5.86–11.34	-4.58–20.79	0.570

Note: p < 0.05 indicates that a publication bias exists.

## Supplementary Materials

Supplementary Materials When searching the Chinese databases, we used Chinese key words and translated them in this article.
