# Supplementary material for: Conventional Treatments plus Acupuncture for Asthma in Adults and Adolescent: A Systematic Review and Meta-Analysis
Source: Evid Based Complement Alternat Med. 2019 Jan 17;2019:9580670. doi: 10.1155/2019/9580670 (PMC6354145; doi:10.1155/2019/9580670)
Supplement: Supplementary Materials — When searching the Chinese databases, we used Chinese key words and translated them in this article. [file 9580670.f1.doc]

Appendix 1

1. Pubmed (133 studies)

(((((((acupunture therapy) OR acupuncture) OR needling therapy) OR needling) OR "Acupunture Therapy"[MeSH]))) AND ((((bronchial asthma) OR asthma) OR asthmatic)) AND ((("Randomized Controlled Trial"[Publication Type] OR "Randomized Controlled Trials as Topic"[MeSH])) OR random*))) Filters: Publication date from 1990/01/01 to 2018/06/01

1. EMBASE (194 studies)

Acupuncture:

'acupunture therapy' OR 'acupuncture'/exp OR 'acupuncture' OR 'needling therapy' OR 'needling' AND [1990-2018]/py

Asthma:

'bronchial asthma'/exp OR 'bronchial asthma' OR 'asthma'/exp OR 'asthma' OR 'asthmatic'/exp OR 'asthmatic' AND [1990-2018]/py

RCT:

random* OR 'randomized controlled trial'/exp OR 'randomized controlled trial' OR 'randomized controlled trial(topic)'/exp OR 'randomized controlled trial(topic)' AND [1990-2018]/py

3. Web of Science (19 studies)

(((TS=(ACUPUNCTURE)) OR (TS=(Needling))) AND ((TS=(bronchial asthma)) OR (TS=(bronchial asthma))) AND (ALL=(1990-2018)) AND (ALL=(randomized controlled trial)) AND (ALL=(Article)))

4. Cochrane Library (85 studies)

#1 MeSH descriptor: [Acupuncture] this term only

#2 acupuncture or needling or acupuncture therapy. ti,ab,kw

#3 #1 OR #2

#4 MeSH descriptor: [undefined] explode all trees

#5 asthma OR bronchial asthma. ti, ab,kw

#6 #4 OR #5

#7 randomized controlled trial

#8 #3 AND #6 AND #7 with Cochrane Library publication date Between Jan 1990 and Jun 2018, in Trials

5. WANFANG (113 studies)

(subject:(acupuncture) + subject:(needling) + subject:(acupuncture treatment)) * subject: ((bronchial asthma) + (asthma))* randomized controlled trial

Publication Date:1990-2018

6. CNKI (698 studies)

SU='acupuncture' OR SU='needling' OR 'acupuncture treatment' AND (SU='bronchial asthma' OR 'asthma')

Filters: Publication date from 1990-01-01 to 2018-06-01

Note: When searching the Chinese databases, we used Chinese key words and translated them in this article.
